# Digital Natives’ Preferences on Mobile Artificial Intelligence Apps for Skin Cancer Diagnostics: Survey Study

**DOI:** 10.2196/22909

**Published:** 2021-08-27

**Authors:** Sarah Haggenmüller, Eva Krieghoff-Henning, Tanja Jutzi, Nicole Trapp, Lennard Kiehl, Jochen Sven Utikal, Sascha Fabian, Titus Josef Brinker

**Affiliations:** 1 Digital Biomarkers for Oncology Group National Center for Tumor Diseases German Cancer Research Center Heidelberg Germany; 2 Department of Dermatology Heidelberg University Mannheim Germany; 3 Skin Cancer Unit German Cancer Research Center Heidelberg Germany; 4 Department of Economics University of Applied Science Neu-Ulm Neu-Ulm Germany

**Keywords:** artificial intelligence, skin cancer, skin cancer screening, diagnostics, digital natives, acceptance, concerns, preferences, online survey

## Abstract

**Background:**

Artificial intelligence (AI) has shown potential to improve diagnostics of various diseases, especially for early detection of skin cancer. Studies have yet to investigate the clear application of AI technology in clinical practice or determine the added value for younger user groups. Translation of AI-based diagnostic tools can only be successful if they are accepted by potential users. Young adults as digital natives may offer the greatest potential for successful implementation of AI into clinical practice, while at the same time, representing the future generation of skin cancer screening participants.

**Objective:**

We conducted an anonymous online survey to examine how and to what extent individuals are willing to accept AI-based mobile apps for skin cancer diagnostics. We evaluated preferences and relative influences of concerns, with a focus on younger age groups.

**Methods:**

We recruited participants below 35 years of age using three social media channels—Facebook, LinkedIn, and Xing. Descriptive analysis and statistical tests were performed to evaluate participants’ attitudes toward mobile apps for skin examination. We integrated an adaptive choice-based conjoint to assess participants’ preferences. We evaluated potential concerns using maximum difference scaling.

**Results:**

We included 728 participants in the analysis. The majority of participants (66.5%, 484/728; 95% CI 0.631-0.699) expressed a positive attitude toward the use of AI-based apps. In particular, participants residing in big cities or small towns (*P*=.02) and individuals that were familiar with the use of health or fitness apps (*P*=.02) were significantly more open to mobile diagnostic systems. Hierarchical Bayes estimation of the preferences of participants with a positive attitude (n=484) revealed that the use of mobile apps as an assistance system was preferred. Participants ruled out app versions with an accuracy of ≤65%, apps using data storage without encryption, and systems that did not provide background information about the decision-making process. However, participants did not mind their data being used anonymously for research purposes, nor did they object to the inclusion of clinical patient information in the decision-making process. Maximum difference scaling analysis for the negative-minded participant group (n=244) showed that data security, insufficient trust in the app, and lack of personal interaction represented the dominant concerns with respect to app use.

**Conclusions:**

The majority of potential future users below 35 years of age were ready to accept AI-based diagnostic solutions for early detection of skin cancer. However, for translation into clinical practice, the participants’ demands for increased transparency and explainability of AI-based tools seem to be critical. Altogether, digital natives between 18 and 24 years and between 25 and 34 years of age expressed similar preferences and concerns when compared both to each other and to results obtained by previous studies that included other age groups.

## Introduction

Deep learning algorithms for image classification play an ever-increasing role in medicine and oncology. Researchers strive to automate and improve the assessment of various diseases, skin cancer in particular, with artificial intelligence (AI) tools. In experimental settings, the performance of AI algorithms achieved accuracies that were on par or even exceeded the results obtained by experienced dermatologists [1-6]. Since early-stage detection of melanoma increases the chances of survival significantly [5], improved AI-based cancer diagnostics might reduce mortality as well as health care expenditure [9-13]. Consequently, an accurate distinction between skin cancer and noncancer through AI-based solutions is of great interest to support diagnosis [3,13,14].

Recent studies about the patient perspective showed that participants expected synergy effects between physical skin examination and the use of mobile apps [15]. The vast majority of participants in another study, with or without previous history of melanoma, had a positive opinion on the use of AI in dermatology, in particular, when used as an assistance system [16]. However, studies have not yet investigated the clear application of AI technology in clinical practice nor the added value for younger user groups.

Here, we report the results of a survey-based study designed to evaluate how and to what extent young adults would be willing to accept AI-based mobile apps for early detection of skin cancer. The general attitudes, preferences, and concerns of potential future skin cancer screening participants below 35 years of age were elaborated, as digital natives may offer the greatest potential for successful implementation of digital assistance systems in clinical practice [15].

## Methods

### Data Collection

We conducted an anonymous online survey using Sawtooth SSI Web Lighthouse Studio 9.8.1. Prior to gathering responses, to ensure comprehensibility and consistency, we tested the survey with 12 volunteers who had no professional background in AI. We then conducted the study with the online survey between March 18, 2020, and April 18, 2020. As we wanted to investigate the preferences and concerns of digital natives, the survey was advertised on three social media channels: Facebook, LinkedIn, and Xing. The survey language was German; the results were translated into English for this paper. Participation was voluntary, and anonymity was ensured by design, to increase the proportion of questions that were answered thoroughly and truthfully.

Participants’ general outlook on using apps for skin cancer examination was collected by asking, “Can you generally imagine covering parts of your skin cancer examination with medical apps?” with response options of “definitely,” “rather yes,” “rather not,” and “definitely not.” Prior experience with health or fitness apps was collected by asking the yes or no question of “Do you use apps to track your health or vital signs?” We included additional questions to obtain sociodemographic data.

To obtain a detailed assessment of the preferences of participants who felt generally positive about mobile AI-based apps (n=484), an adaptive choice-based conjoint (ACBC) was integrated into the survey. Based on the insights from our preliminary qualitative research, 7 app features and corresponding level options were developed for this investigation (Multimedia Appendix 1). Moreover, to ensure that no unrealistic combinations were presented, certain prohibitions were specified (Multimedia Appendix 2).

The ACBC process typically consists of 3 parts. First, in the so-called build-your own section, participants created their own customized product and familiarized themselves with the relevant app features as well as the corresponding levels. In the next part—the screening section—apps with specific feature combinations were presented, and participants were asked whether they would consider using app versions with these combinations. Finally, within a choice tournament, participants were asked to choose their preferred product from a range of apps based on their answers in the previous parts.

For evaluation of frequently cited concerns about AI-based tools, a maximum difference scaling (MaxDiff) section was included in the survey with a special focus on participants that generally refused the use of mobile skin examination tools (n=244). Based on the insights from preliminary qualitative research as well as literature review [16,17], 6 inhibitory aspects were selected for the analysis. Participants were shown several subsets of possible concerns and were asked to specify which one they considered the most and the least important. Participants made choices rather than expressing their strength of concerns with a numerical or rating scale. In this way, greater discrimination could be achieved and a comparison of the relative impact of participants’ concerns was possible. Moreover, to ensure that no relevant issues were left out, participants received the opportunity to express additional concerns in a free-text question.

### Data Validation

A total of 1548 participants below 35 years of age took part in the survey. To ensure data quality for subsequent analysis, responses that did not fulfill our internal quality criteria were identified and eliminated from the data set. For this purpose, a multilevel data cleaning process was applied. We excluded participants who answered only part of the questionnaire (n=731), participants living outside of Germany (n=36), and participants under the age of 18 (n=21). Furthermore, contradictions in the participants’ answers were examined; as a result, we removed another 2 participants. To minimize the risk of including participants who did not consider the topic seriously or possibly interrupted the survey, participants that answered the survey extremely fast or slow were left out [18,19]. After deleting responses of participants that took less than 2 minutes (n=15) or more than 60 minutes (n=15), a validated data set of 728 participants remained.

### Data Analysis

To evaluate participants’ general attitudes toward mobile apps for early detection of skin cancer, a descriptive analysis was conducted. The categories “definitely” and “rather yes” were summarized as a positive attitude while “rather not” and “definitely not” were summarized as a negative attitude toward mobile apps for skin examination. Statistical analysis was performed using SPSS, version 25.0 (IBM Corporation). Chi-square tests were performed to outline associations between sociodemographic characteristics and selected items of the questionnaire. We therefore conducted prespecified subgroup analyses on gender, residence, type of insurance, and prior experience with health or fitness apps. In the results section, we report only significant differences with a significance level set to *P*<.05 for all analyses. We computed 95% CIs for the main results using the normal distribution approximation.

ACBC data were analyzed using hierarchical Bayes estimation. The results were expressed in terms of counts, importance values, and utilities [20-22]. Count analysis examined how often certain levels were defined as unacceptable or must-have criteria within the screening section of the ACBC [22]. To evaluate the relevance of an attribute within the choice process of participants, average importance values were calculated [20,21]. Thus, for each feature, the utility value of the level that was regarded as most useful minus the level that was considered least useful represented the utility range (X). Subsequently, all utility ranges were summed (Y), and the share of each feature was determined based on the equation:

Feature importance (%) = (X/Y) × 100.

We calculated 95% CIs, taking the average feature importance score ±1.96 × SE. SE was computed by taking the SD of the importance score divided by the square root of the sample size. Part-worth estimation was performed to determine which feature levels were preferred from the participants’ point of view. Utility values are presented as zero-centered differences within each feature.

MaxDiff data were expressed as sample mean scores and then rescaled to probability scores that reflect the likelihood that a concern was selected as “most important” within MaxDiff. We calculated 95% CIs, taking the average rescaled probability score ±1.96 × SE.

## Results

### Baseline Characteristics of the Study Sample

The demographic characteristics of the study sample are shown in Table 1. The median age was 24 years and the age distribution was fairly symmetrical.

**Table 1:** Baseline characteristics of the study sample.

Sociodemographic characteristics		Values, n (%)
**Gender**		
	Female	523 (71.8)
	Male	205 (28.2)
**Residence**		
	Large cities (>100,000 inhabitants)	218 (29.9)
	Small towns (10,000-100,000 inhabitants)	222 (30.5)
	Rural areas (<10,000 inhabitants)	288 (39.6)
**Type of insurance**		
	Private insurance	82 (11.3)
	Public insurance	646 (88.7)
**Prior experience with health/fitness apps**		
	Yes	246 (33.8)
	No	482 (66.2)
**Age (years)**		
	18-24	420 (57.7)
	25-34	308 (42.3)

### General Attitude Toward Mobile Apps for Early Detection of Skin Cancer

**Figure 1:**
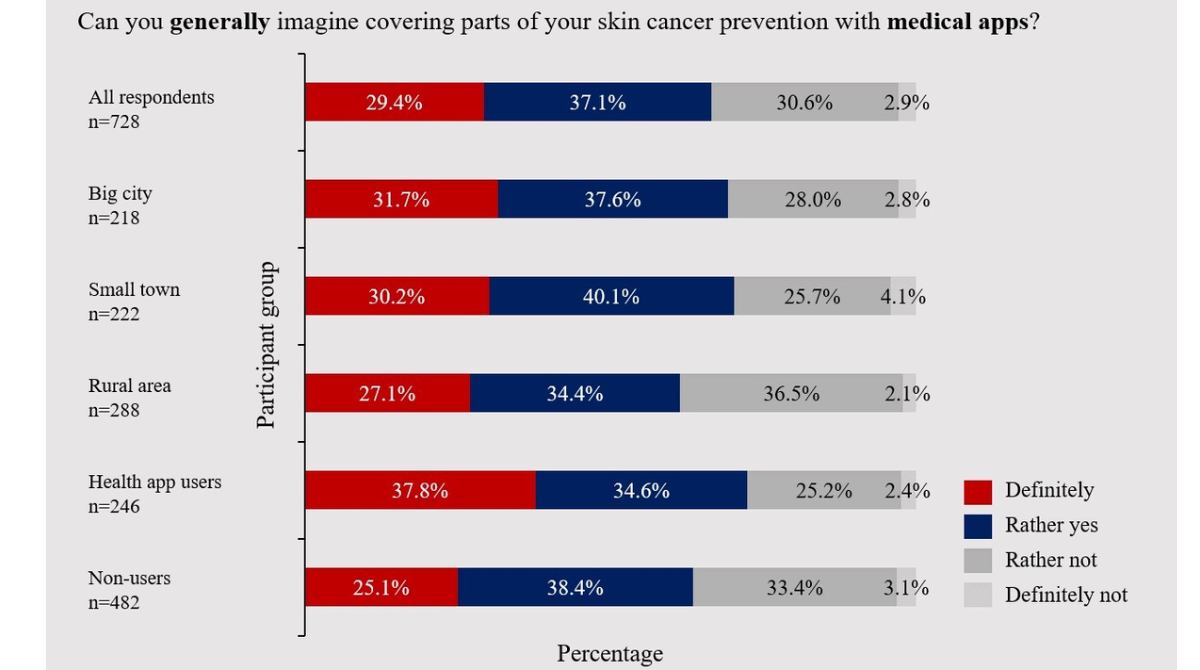
Attitudes toward the use of mobile artificial intelligence–based apps for skin examination. Bar chart depicts the distribution of participants’ general attitude depending on prespecified sociodemographic characteristics.

Of all included participants (n=728), 484 participants (66.5%; 95% CI 0.631-0.699) were positive-minded toward the use of mobile apps for early detection of skin cancer. Only 21 participants explicitly ruled it out altogether (Figure 1). When comparing the age classes of 18 to 24 years and 25 to 34 years, no significant difference was observed (*P*=.97). Subgroup analysis revealed significant differences based on prespecified sociodemographic criteria. Out of the 440 participants residing in small towns or big cities, 307 (69.8%; 95% CI 0.655-0.740) felt significantly more positive toward mobile skin cancer screening apps than participants living in rural areas (177/288, 61.5%; 95% CI 0.558-0.671; *P*=.02). Moreover, previous experience with health or fitness apps had a significant effect on the willingness to use medical apps for early detection of skin cancer (*P*=.02).

Of all participants that were generally negative-minded toward mobile apps for skin examination (n=244), 145 participants (59.4%; 95% CI 0.553-0.656) would rather consider using AI-based solutions if they received a reduced contribution from their health insurance company. In this context, participants between 18 and 24 years of age (94/141, 66.7%; 95% CI 0.589-0.745) were significantly more receptive to financial incentives than participants between 25 and 34 years of age (51/103, 49.5%; 95% CI 0.399-0.592; *P*=.007).

### Preferences of the Future Generation of Skin Cancer Screening Participants

#### Must-Have and Unacceptable Criteria

For successful development of patient-usable AI systems, the identification of must-have and unacceptable criteria provides meaningful insights. The ACBC analysis identified app features that would cause future screening participants to reject the app. Of all participants that were generally positive-minded toward mobile apps for skin examination (n=484), 99 participants (20.5%; 95% CI 0.169-0.241) stated that they were not willing to rely on an exclusively app-based diagnosis. On the other hand, 179 participants of the 484 (37.0%; 95% CI 0.327-0.413), completely ruled out app versions with an accuracy of ≤65%. Data storage without encryption represented an exclusion criterion for 155 of the 484 participants (32.0%; 95% CI 0.279-0.362). Moreover, 100 of the 484 participants (20.7%; 95% CI 0.171-0.243) generally rejected apps without background information about the decision-making process. In line with this result, a further 96 participants of the 484 (19.8%; 95% CI 0.163-0.234) specified a basic explanation of the reasoning for the decision as an absolute must-have criterion.

#### Relative Importance of Individual App Features

Importance, on the other hand, indicates which relevance a certain app feature exerts on the decision-making process of participants [20,21]. Within this study, the app features “accuracy of the app,” “field of application,” and “data storage,” on average, bore the greatest relative importance for participants’ selection; the “accuracy of the app” constituted the top priority across all prespecified subgroups (Table 2). In contrast, “data processing” and “data usage” exerted only a minor influence on the choice of medical apps for skin cancer detection, thus opening up opportunities to pursue research interests without endangering the acceptance of future screening participants. Subgroup analysis on gender revealed that women attached considerably more importance to the app feature “explainability of the results” (females: 14.1%, males: 11.4%).

**Table 2:** Average relative importance of individual app features within ACBC.

Feature	Average importance, % (95% CI)
Accuracy of the app	20.4 (19.6-21.3)
Field of application	17.6 (16.9-18.3)
Data storage	17.4 (16.5-18.2)
Receipt of diagnosis	15.0 (14.2-15.7)
Explainability of the results	13.3 (12.7-14.0)
Data usage	9.8 (9.3-10.3)
Data processing	6.5 (6.2-6.8)

#### Preferred Levels of Individual App Features

After the relevance of individual app features within the process of choosing apps was examined, a detailed analysis was performed to determine which level of each app feature offers the greatest perceived usefulness from the participants’ point of view [20] (overview of all app features and the corresponding levels, see Multimedia Appendix 1). This step is generally known as part-worth estimation.

Unsurprisingly, part-worth estimation revealed that the higher the accuracy level, the higher the added value from the participants’ perspective. However, improving the accuracy from 85% to 90% disproportionately raised the perceived benefit (from 28.0 to 56.7), while an increase from 80% to 85% triggered only slight advancement in utility value (from 21.6 to 28.0). Consequently, achieving an accuracy of 80% might not lead to major shifts in the number of individuals willing to use a skin cancer screening app. The achievement of an accuracy of 90%, on the other hand, represented a convincing argument for the vast majority.

Concerning the feature “data usage,” an integration of additional clinical information, such as age, gender, and patient medical history provided the greatest benefit for participants. Moreover, participants did not mind their data being used for research purposes in an anonymous way and, therefore, explicitly favored anonymous data processing for future research projects. These preferences were reflected regardless of sociodemographic characteristics across all prespecified subgroups.

Participants preferred an app scenario where the “field of application” is limited to appointment prioritization, followed by a personal consultation with a specialist. Moreover, participants favored that their health data be stored encrypted in the app and additionally protected by a personal password. Participants living in big cities would even prefer not to store their data at all, rather than storing them encrypted without personal password protection. The delivery of diagnostic results in real time constituted the preferred level regarding the receipt of diagnosis. The more detailed the explanation of the decision-making process, the higher the perceived usefulness was for participants.

### Concerns of the Future Generation of Skin Cancer Screening Participants

Analyzing the MaxDiff data of this survey, no single criterion stood out significantly (Table 3). In terms of the probability scores, privacy concerns (24.3), insufficient trust in the app (23.5), and a lack of personal interaction (21.7) represented the dominant barriers from the participants’ point of view. Moreover, concerns about incorrect app usage played a considerable role (17.7). In contrast, frequently mentioned aspects, such as the effort to deal with the functionality of the app (6.8) or the lack of technical affinity (6.0), exerted only minor influences.

**Table 3:** Evaluation of frequently cited concerns according to hierarchical Bayes estimation.

Ranking according to hierarchical Bayes estimation	Rescaled score^a^ (95% CI)
Privacy concerns	24.3 (22.3-26.2)
Insufficient trust in the app	23.5 (22.1-25.0)
Lack of personal interaction	21.7 (20.0-23.5)
Incorrect app usage	17.7 (16.1-19.2)
Effort to deal with the functionality	6.8 (5.7-7.9)
Lack of technical affinity	6.0 (5.0-7.1)

^a^The rescaled score ranged from 0 to 100.

A total of 99 additions were made as part of the free-text entry. All responses were screened and analyzed in a qualitative manner. However, the majority of the answers had already been covered by the 6 selected main aspects, either by repeating or concretizing them, using examples. Beyond that, participants mentioned concerns regarding discrimination of elderly participants without smartphone experience, costs for both mobile devices and apps, and potential psychological burdens in case of suspected cancer.

## Discussion

### Principal Results

The majority of the future generation of skin cancer screening participants were ready to accept mobile apps for early detection of skin cancer. However, participants stated certain unacceptable and must-have criteria that need to be considered when developing patient-oriented AI-based solutions for dermatology. For translation into clinical practice, the demand for increased transparency and explainability appears particularly critical. Within the current state of AI, it is not possible to fully explain the reasoning of the decision making due to the black box phenomenon [23-26]. Therefore, to achieve broad acceptance among screening participants, approaches that encourage at least a basic explanation of the decision-making process are required.

The attributes “data processing” and “data usage” exerted only a minor influence on the choice of medical apps for skin examination; in fact, participants explicitly supported anonymous data processing and would therefore likely support the concept of open data, which encourages the sharing and release of data sets across research and clinical institutions. Moreover, from the participants’ point of view, there were no reservations against the inclusion of clinical information such as age, gender, and patient medical history.

For successful implementation into clinical practice, concerns of skeptical participants as well as identified rule-out criteria must be considered. Against this backdrop, the proper way to incorporate AI solutions within dermatology is by augmenting human intelligence and not replacing it. To leverage the potential of AI-based assistant systems, future research and clinical projects should emphasize personal interaction while simultaneously accomplishing a synergy between humans and AI systems. This approach coincides with the preferences of the majority of participants who were positive-minded toward the use of mobile apps for skin cancer detection in this ACBC, as well as with results obtained by previous studies [15,16]. Moreover, the active promotion of participants’ ability to act constitutes a key aspect. Mobile AI apps can only reach their full potential if future screening participants receive guidance and decision support. For individuals to trust Al-based apps, both orientation points and reliable and comprehensible health information are required. In this way, patients get an indication of how to distinguish potential medical AI assistance from conventional fitness or health apps. Furthermore, evaluation of participant concerns highlighted the demand for standardized regulations on how data are stored and protected within AI-based apps. This demand is also driven by the fact that 155 of 484 participants (32.0%) stated that data storage without encryption is an absolute exclusion criterion. Consequently, to achieve patient-oriented apps for dermatology, data security must play a key role within the whole development process.

### Limitations

The baseline characteristics of this study sample showed that participants were predominantly female, thus not representative of the gender distribution in the general population. Since participants were recruited through social media, there is a risk of sampling bias, as social media users may be more likely to use mobile apps. Therefore, the results that we obtained are probably not fully generalizable to the general population of digital natives.

Importance was directly affected by the range of levels selected for each app feature as well as the total number of features [20,21]. Adding or removing a very popular or unpopular level to a feature would change the importance of all other attributes. Consequently, this paper could only reflect the importance relative to the features that were tested within this ACBC design.

MaxDiff, by definition, involves only comparative judgments. Thus, this elaboration cannot draw conclusions about the absolute magnitude of the selected impeding factors. One way to further increase the information value is to integrate additional questions that deliver an anchoring point (eg, specify the importance of one item), so that information in an absolute sense could be obtained [27].

### Conclusions

The majority of potential screening participants below 35 years of age were ready to accept AI-based solutions. However, participants’ demands for increased transparency and explainability of AI-based tools must be considered for successful translation into clinical practice. Digital natives between 18 and 24 years and between 25 and 34 years of age showed similar preferences and concerns when compared to each other as well as to other age groups. They preferred the use of AI-based solutions as expert assistance systems, attached considerable value to the accuracy of AI apps, and expressed data privacy concerns.

## Appendix

Multimedia Appendix 1 Overview about app features and the corresponding levels for this ACBC investigation.

Multimedia Appendix 2 Prespecified prohibitions of this ACBC study.

## Abbreviations

ACBC: adaptive choice-based conjoint
AI: artificial intelligence
MaxDiff: maximum difference scaling
