# Supplementary material for: Digital Natives’ Preferences on Mobile Artificial Intelligence Apps for Skin Cancer Diagnostics: Survey Study
Source: JMIR Mhealth Uhealth. 2021 Aug 27;9(8):e22909. doi: 10.2196/22909 (PMC8433862; doi:10.2196/22909)
Supplement: Multimedia Appendix 1 [file mhealth_v9i8e22909_app1.docx]

# MULTIMEDIA APPENDIX

Multimedia Appendix 1: Overview about app features and the corresponding levels for this ACBC investigation.

| **Field of Application** |  |
| --- | --- |
|  | Diagnosis exclusively via app |
|  | Data collection via app with subsequent online diagnosis by experts |
|  | Appointment prioritization via app, followed by a personal visit with a specialist |
| **Data Usage** |  |
|  | Diagnosis exclusively based on image input |
|  | Information about age and gender is included. |
|  | Information about age, gender and patient medical history is included. |
| **Data Storage** |  |
|  | Data are not stored in the app. |
|  | Data are stored encrypted in the app and protected by personal password. |
|  | Data are stored encrypted in the app. |
|  | Data are stored in the app without encryption. |
| **Data Processing** |  |
|  | Data are used exclusively for diagnosis. |
|  | Data are used for diagnosis and anonymized for research purposes. |
|  | Data are used for diagnosis and for research purposes. |
| **Explainability of the Results** |  |
|  | No background information about the decision-making process |
|  | Basic explanation of the decision-making process |
|  | Detailed explanation of the decision-making process |
| **Accuracy of the App (in %)** |  |
|  | 65 percent |
|  | 70 percent |
|  | 75 percent |
|  | 80 percent (comparable with experienced dermatologists) |
|  | 85 percent |
|  | 90 percent |
| **Receipt of Diagnosis** |  |
|  | Real time delivery |
|  | Within 24 hours |
|  | Within two to three days |
|  | Within four to seven days |
|  | Within eight to 21 days |
|  | After more than 21 days |
