# Supplementary material for: Digital Natives’ Preferences on Mobile Artificial Intelligence Apps for Skin Cancer Diagnostics: Survey Study
Source: JMIR Mhealth Uhealth. 2021 Aug 27;9(8):e22909. doi: 10.2196/22909 (PMC8433862; doi:10.2196/22909)
Supplement: Multimedia Appendix 2 [file mhealth_v9i8e22909_app2.docx]

# MULTIMEDIA APPENDIX

Multimedia Appendix 2: Prespecified prohibitions of this ACBC study.

| **Prohibitions** | |
| --- | --- |
| Diagnosis exclusively via app | Receipt of diagnosis within 24 hours or later |
| Data collection via app with subsequent online diagnosis by experts | Receipt of diagnosis in real time |
| Appointment prioritization via app, followed by a personal visit with a specialist | Receipt of diagnosis within 24 hours or earlier |
